# Immunoprevention of triple-negative breast cancer with a novel multivalent vaccine

**DOI:** 10.3389/fimmu.2025.1638526

**Published:** 2025-09-03

**Authors:** Sang Beom Lee, Jianfei Qian, Jing Pan, Shizuko Sei, Yian Wang, Ming You

**Affiliations:** ^1^ Center for Cancer Prevention, Houston Methodist Cancer Center, Houston Methodist Research Institute, Weill Cornell College of Medicine, Houston, TX, United States; ^2^ Center for Translational Research in Hematological Malignancies, Houston Methodist Neal Cancer Center, Houston Methodist Research Institute, Weill Cornell College of Medicine, Houston, TX, United States; ^3^ Chemopreventive Agent Development Research Group, Division of Cancer Prevention, National Cancer Institute, Bethesda, MD, United States

**Keywords:** triple-negative breast cancer (TNBC), topoisomerase 2 alpha (TOP2A), hypoxia inducible factor-1α (HIF-1α), insulin like growth factor-1 receptor (IGF-1R) peptide vaccine, immunoprevention, multi-antigen multi-peptide (TNBCvax) vaccine

## Abstract

Triple-negative breast cancer (TNBC) is associated with a poor prognosis due to high recurrence rates and a lack of targeted therapies. Significant challenges in developing efficacious TNBC cancer vaccines are tumor antigen heterogeneity and the risk of antigen-negative variant escape, where target antigen-negative tumor cells can emerge, evading single-antigen vaccine-induced immunity, and drive tumor growth. To address this, we developed TNBCvax, a multi-antigen, multi-peptide vaccine targeting three tumor-associated antigens overexpressed in TNBC: TOP2A, HIF-1α and IGF-1R. The immune preventive effect of TNBCvax was evaluated in both a syngeneic M6 TNBC tumor graft model and the C3(1)/Tag genetically engineered mouse model of TNBC. Our findings demonstrate that TNBCvax significantly reduced tumor development and progression, compared to single-antigen vaccines. TNBCvax induced a robust tumor-associated antigen-specific immune response as evidenced by the increased infiltration of CD3+ T cells, particularly CD8+ T cells, with elevated levels of granzyme B and tumor necrosis factor alpha (TNF-α). TNBCvax was well-tolerated with no significant major organ toxicities, supporting its potential safety in the clinic. In conclusion, TNBCvax offers a promising immunopreventive strategy against TNBC by targeting multiple antigens to provide a broader and more robust immune coverage against TNBC antigens while reducing the risk of antigen-negative variant escape.

## Introduction

Breast cancer is the most common malignancy among women. In 2025, an estimated 316,950 new cases of invasive breast cancer are expected to be diagnosed in women in the U.S., alongside 59,080 new cases of ductal carcinoma *in situ* (DCIS) (1). Triple-negative breast cancer (TNBC) is an aggressive and highly heterogeneous subtype, characterized by the absence of estrogen receptor (ER), progesterone receptor (PR), and human epidermal growth factor receptor 2 (HER2) expression (2). TNBC accounts for approximately 15-20% of all breast cancer cases and is associated with a poorer prognosis compared to other subtypes, primarily due to its high recurrence rate and limited treatment options. The absence of targeted therapies for TNBC underscores the urgent need for innovative preventive strategies (3).

Recent advances in cancer immunotherapy, particularly in the development of cancer vaccines, have shown promise in preventing tumor development by inducing robust immune responses against tumor-associated antigens (4–8). However, the genetic and phenotypic diversity of TNBC poses significant challenges to single-antigen vaccines, primarily due to antigen-negative variant escape, which can render single-antigen vaccines less effective over time. Evidence from previous studies, including DCIS vaccine trials, has demonstrated that targeting a single antigen, such as HER2, can lead to the emergence of antigen-loss variants, ultimately compromising vaccine efficacy (9, 10).

We developed TNBCvax, a multi-antigen, multi-peptide vaccine designed to simultaneously target three key tumor-associated antigens: TOP2A, HIF-1α, and IGF-1R. Building on our previous study for TOP2A vaccine (12), TNBCvax combines three distinct antigens to enhance antitumor immunity, address TNBC heterogeneity, and lower the risk of immune escape. Each of these antigens plays a critical role in TNBC pathogenesis and is overexpressed in most TNBC cases. TOP2A, a DNA topoisomerase involved in DNA replication, is frequently overexpressed in TNBC and has been identified as a potential immunotherapeutic target (11). Our earlier, a TOP2A vaccine demonstrated high efficacy in preventing TNBC in preclinical models, reducing tumor incidence and tumor burden while also inducing a strong and specific immune response (12). These promising results provided the foundation for further exploration of multi-antigen strategies for TNBC prevention.

HIF-1α, a transcription factor that regulates cellular response to hypoxia, is overexpressed in over 80% of TNBC cases, and is considered a potential universal antigen for this subtype (13). IGF-1R, the insulin-like growth factor receptor, is implicated in cell survival and proliferation, with overexpression observed in 87% of primary breast tumors including TNBC, and its phosphorylated form correlates with poor clinical outcomes (14, 15). Previous work by Dr. Nora Disis and colleagues demonstrated the efficacy of MHC class II-restricted vaccines targeting HIF-1α and IGF-1R in preclinical models (16). Incorporating these three antigens into TNBCvax was intended to provide complementary immune targeting and improve long-term tumor control.

In this study, we evaluated the immunogenicity, antitumor efficacy, and immunological mechanisms of TNBCvax in pre-clinical TNBC models. Our findings suggest that TNBCvax effectively inhibited the development of mammary tumors by multi-epitope-specific Th1 immunity, enhances CD8+ T cell–mediated cytotoxic responses, and fosters central memory T cell formation, leading to improved tumor control without significant toxicity.

## Methods

### Mice

Transgenic C3(1)/Tag mice and C3(1)/Tag-REAR (rearrangement) mice were a generous gift from Dr. Jeffery E. Green. FVB/N wild type mice were purchased from the Jackson Laboratory. For all experiments, only F2 generation C3(1)/Tag or C3(1)/Tag-REAR mice were used. Mice were randomly assigned to treatment groups prior to vaccination. Group sizes (n) for each experiment are reported in the figure legends. All mice were maintained and bred in the Comparative Medicine Program at the Houston Methodist Research Institute, Houston, TX. All procedures were approved by the Institutional Animal Care and Use Committee (IACUC).

### Cell lines

The M6 tumor cell line, derived from C3(1)/Tag mice, were provided by Dr. Jeffery E. Green. The cell lines were maintained in DMEM medium containing high glucose (Gibco), supplemented with 5% fetal bovine serum, penicillin/streptomycin, and sodium pyruvate (Invitrogen).

### Immunohistochemistry

IHC staining was performed by the Research Histology Core at Houston Methodist Research Institute. Mouse mammary gland samples from C3(1)/Tag mice were formalin-fixed and paraffin-embedded (Sakura Tissue Tek VIP5). Fixed mammary gland tissues were further processed with IHC staining with anti‐HIF-1α (Invitrogen, # PA1-16601), anti‐IGF-1R (abcam,ab39398) or anti-TOP2A (Invitrogen, #PA5-110707) primary antibodies. Slides were applied with enzyme conjugate secondary anti‐mouse antibody, followed by adding DAB substrate‐chromogen mixture.

### ELISPOT assay

Cell suspensions from whole spleens were filtered through a 70 μm cell strainer (BD) and subjected to red blood cell lysis using ACK lysis buffer. 4.0 x 10^5^ cells were plated into individual wells of a MAIPS4510 multiscreen 96-well plate coated with anti-interferon γ (IFN-γ) and anti-IL-10 detection antibody and containing media with either peptide, concanavalin A (positive control), HIV peptide (negative control), or no antigen (negative control). After 72-hour incubation, plates were washed and incubated with a secondary antibody (BD) overnight at 4 °C. Wells were then washed with PBS and HRP streptavidin was added. Following 1-hour incubation, the plate was developed using AEC substrate for between five to 25 minutes. An automated plate reader system (CTL Technologies) was used to image the plates and quantify spot numbers.

### Vaccine preparation and immunization

**Figure 1:**
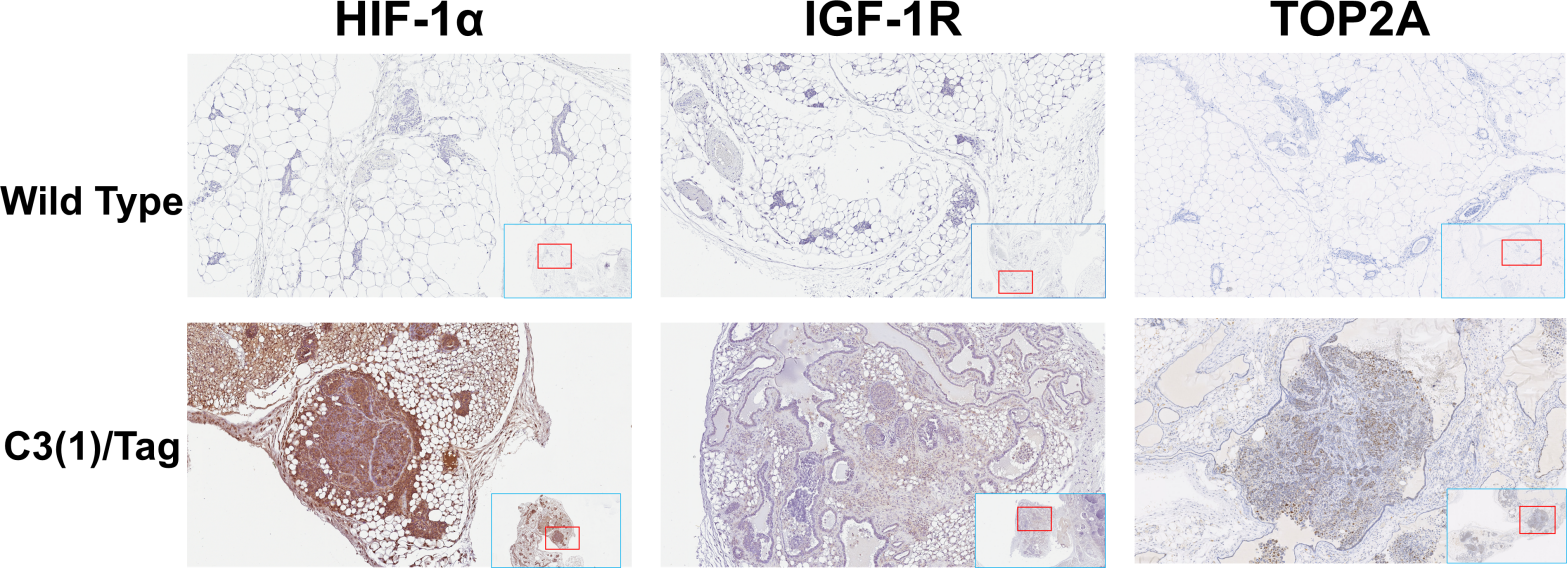
Overexpression of the HIF-1α, IGF-1R and TOP2A in mouse TNBC tissues. Representative images of immunohistochemistry staining with HIF-1α, IGF-1R and TOP2A on the mammary gland tissues of 16 weeks old C3(1)/Tag and wildtype mice. All images were scanned and captured with Aperio ImageScope (Leica Biosystems, IL).

**Figure 2:**
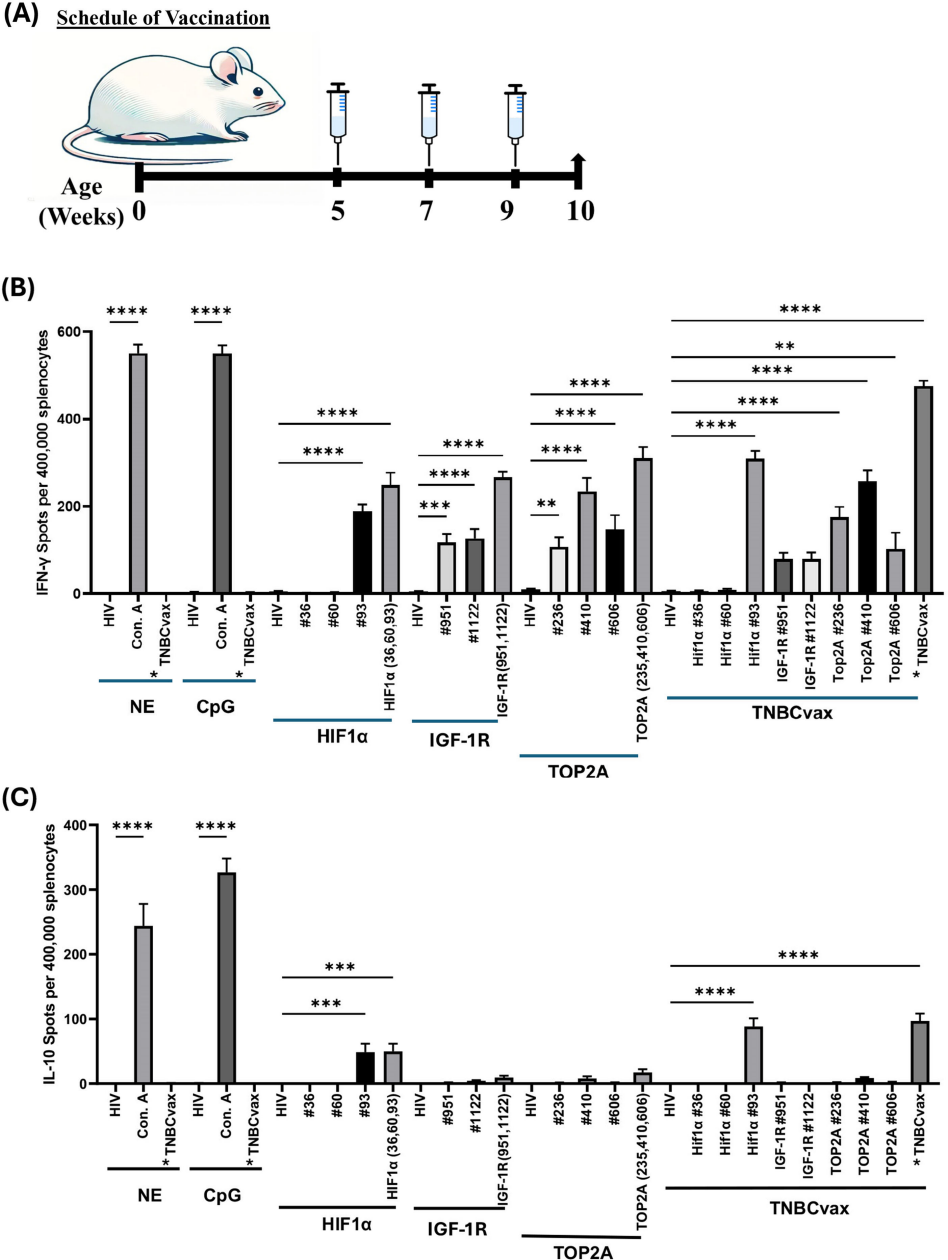
Immunogenicity of single *vs*. multi-antigen, multi-peptide (TNBCvax) vaccines in wild-type non-tumor bearing mice. **(A)** Vaccination schema of the experiments on C3(1)/Tag mice. The study included six vaccinated groups: the NE group, which received PBS injections only; the CpG-only group, which received the adjuvant CpG alone; the HIF-1α, IGF-1R, and TOP2A groups, each vaccinated with peptides specific to their respective antigens; and the TNBCvax group, which received vaccination with peptides targeting all antigens.**(B)** IFN-γ based ELISPOT assay results. Representative IFN-γ based ELISPOT assay results showing T cell responses to specific peptides from mouse splenocytes. **(C)** IL-10 based ELISPOT assay. Splenocytes were collected from vaccinated mice and pulsed with negative control peptide (HIV peptide), positive control (Concanavalin A, Con. A), each antigen peptide (HIF-1α, IGF-1R and TOP2A) or mixed 8 peptides (TNBCvax). After 72h of incubation, the ELISPOT assay was performed, plates were scanned, and spot numbers were statistically analyzed. *TNBCvax refers to a mixture of all peptides (TOP2A, HIF-1α, and IGF-1R). n=5 mice per group were analyzed for ELISPOT assays. Data are shown as the mean ± SEM, One-way ANOVA, ** p<0.01, *** p<0.001, **** p<0.0001.

**Figure 3:**
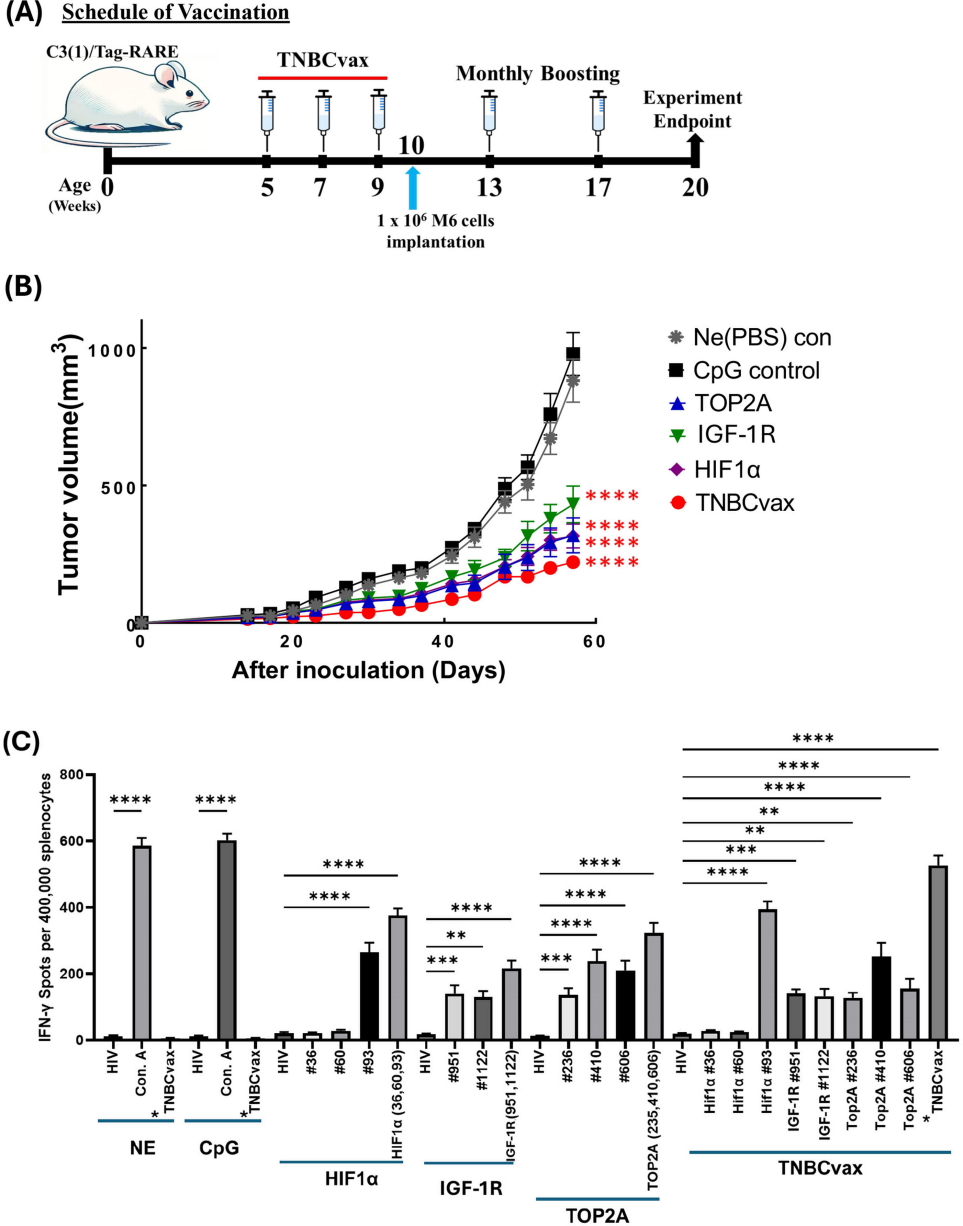
TNBCvax vaccination inhibited tumor growth in the syngeneic C3(1)/Tag-REAR mouse model. **(A)** Experimental design and timeline of vaccine administration. **(B)** Tumor growth curves after M6 tumor cell inoculation. The study included six vaccinated groups: the NE group (n=14), which received PBS injections only; the CpG-only group (n=14), which received the adjuvant CpG alone; the HIF-1α (n=14), IGF-1R (n=14), and TOP2A groups (n=13), each vaccinated with peptides specific to their respective antigens; and the TNBCvax group (n=14), which received vaccination with peptides targeting all antigens. Following implantation, tumor diameters were measured using a digital caliper and tumor volumes were calculated using the formula: maximum diameter × (minimum diameter)^2^ × 0.5. Data are shown as the mean ± SEM, One-way ANOVA at 57 days, **** p<0.0001. **(C)** IFN-γ based ELISPOT assay result in the syngeneic C3(1)/Tag-REAR mouse model. Splenocytes were collected from vaccinated mice and pulsed with negative control peptide (HIV peptide), positive control (Concanavalin A, Con. A), each antigen peptide (HIF-1α, IGF-1R and TOP2A) or mixed 8 peptides (TNBCvax). After 72h of incubation, the ELISPOT assay was performed, plates were scanned, and spot numbers were statistically analyzed. *TNBCvax refers to a mixture of all peptides (TOP2A, HIF-1α, and IGF-1R). n=6 mice per group were analyzed for ELISPOT assays. Data are shown as the mean ± SEM, One-way ANOVA, ** p<0.01, *** p<0.001, **** p<0.0001.

The TNBCvax peptide vaccine comprises three antigens and eight peptides targeting distinct regions of HIF1α, IGF-1R and TOP2A. Peptides were selected based on predicted high-affinity binding to MHC class II molecules (NetMHCIIpan/IEDB), murine–human sequence similarity, and previously demonstrated preclinical immunogenicity. HIV-derived peptide was used as an irrelevant negative control in ELISPOT assays to exclude non-specific antigen responses. TOP2A vaccines are designed by optimal binding affinity to MHC II were identified using a combined scoring system (12). Dr. Nora Disis’ group has developed HIF-1α (16) and IGF-1R (5) MHC-II vaccines. The sequences of all peptides and their similarity between mouse and human are provided in **Table 1**. Mice were administered a total dose of 400 μg of TNBCvax, with each peptide contributing 50 μg to the formulation. All peptides were procured from Genemed Synthesis and were diluted in phosphate-buffered saline (PBS) to a final volume of 50 μl per mouse. The vaccine formulation was supplemented with an equal amount of the adjuvant CpG (Class B CpG oligonucleotide, a murine TLR9 ligand; Cat. No. tlrl-1826, InvivoGen), which was also administered at 50 μg per mouse (18–20). The final vaccine volume per mouse was adjusted to 100 μl. Subcutaneous injections were performed according to the timelines depicted in **Figures 1A**, **2A**, and **3A**.

**Table 1.** Amino acid sequences of TOP2A, HIF-1α, and IGF-IR vaccines.

Antigen	Amino Acid Sequences		Homology vs Mouse
TOP2A	#236	KDIVALMVRRAYDIA	100%
	#410	ILNWVKFKAQVQLNKK	100%
	#606	KKWKVKYYKGLGTSTSK	100%
HIF-1α	#38	VSSHLDKASVMRLTIS	100%
	#60	LLDAGGLDSEDEMKAQMDCFYLK	83%
	#93	DDGDMVYISDNVNKYMGLTQFELAG	92%
IGF-IR	#951	KDIVALMVRRAYDIA	100%
	#1122	ILNWVKFKAQVQLNKK	100%

### *In vivo* tumorigenicity assay

**Figure 4:**
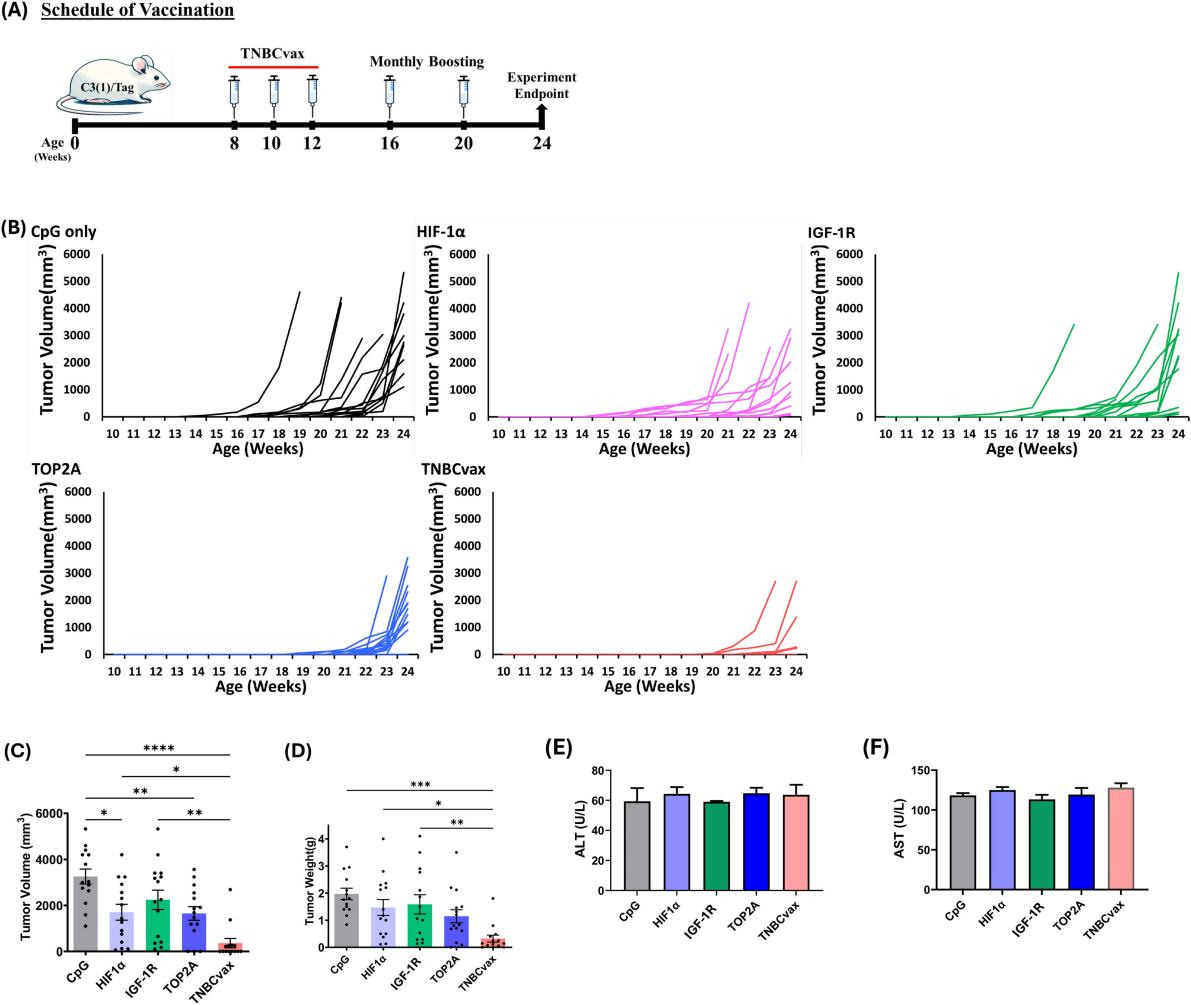
The anti-tumor efficacy of TOP2A, HIF-1α, and IGF-1R single-antigen vaccines and TNBCvax on TNBC in C3(1)/Tag transgenic mice. **(A)** Experimental design and timeline of vaccine administration. **(B)** Tumor growth in C3(1)/Tag transgenic mice. The study included six vaccinated groups: the CpG group, which received the adjuvant CpG alone; the HIF-1α, IGF-1R, and TOP2A groups, each vaccinated with peptides specific to their respective antigens; and the TNBCvax group, which received vaccination with peptides targeting all antigens. n=14–15 mice per group for tumor growth analysis. **(C)** Tumor volumes. Palpable tumor diameters were measured at 24-weeks of age. Data are shown as the mean ± SEM, One-way ANOVA, *p < 0.05, ** p<0.01, **** p<0.0001. **(D)** Weight of each tumor was taken at the time of euthanization. One-way ANOVA, * p < 0.05, ** p<0.01, *** p<0.001. **(E–F)** ALT and AST levels at 24-weeks of age. The toxicity study included six vaccinated groups: CpG group (n=5), HIF-1α (n=5), IGF-1R (n=5), TOP2A groups (n=5), and TNBCvax group (n=5).

For a syngeneic tumor graft model, C3(1)/Tag-REAR mouse line derived from a C3(1)/Tag founder line and a syngeneic M6 mammary tumor line were used as previously reported (21). C3(1)/Tag-REAR mice have no spontaneous cancer phenotype due to the loss of one copy (or several) of the C3(1)/Tag-antigen transgene (22). M6 cells, derived from a C3(1)/Tag transgenic mammary tumor, were implanted into the mammary fat pad of C3(1)/Tag-REAR mice. M6 cells were washed, resuspended in PBS at a density of 1 x 10^6^ cells in 100 μl PBS, and injected into the #4 mammary fat pads of female C3(1)/Tag-REAR mice. Following implantation, tumor diameters were measured using calipers, and tumor volumes were calculated using the formula: maximum diameter × (minimum diameter)^2^ × 0.5. In the spontaneous GEM model, C3(1)/Tag mice were treated with the peptide vaccine following the experimental design in **Figures 4A**. C3(1)/Tag mice were euthanized at 24 weeks of age for estimation of tumor development. Tumor volumes were measured similarly as described above.

### Flow cytometry

For tumor immune profiling, mammary gland tumors from mice were harvested at the end of study, minced into 1–2 mm pieces, digested at 37 °C for 30 minutes with mouse tumor dissociation buffer (Miltenyi Biotec, CA), and passed through a 40 μm nylon mesh to generate single-cell suspensions. Cells were incubated with surface markers of interest at the recommended or titrated concentrations, incubated at 4°C for 30 minutes, and protected from light. After incubation, cells were washed and resuspended in FACS fixation buffer for either analysis or intracellular staining. To begin intracellular staining, cells were fixed with Foxp3/Transcription factor staining buffer (eBioscience) and stained with intracellular markers of interest at the recommended or titrated concentrations at 4°C for at least 30 minutes while protecting them from light. Samples were washed with permeabilization buffer and resuspended in FACS fixation buffer. Stained cells were fixed in 1% paraformaldehyde and permeabilized following the manufacturer’s instructions to evaluate the expression of intracellular targets, granzyme B, IFN-γ and TNF-α. Flow cytometry was conducted using an LSR-II flow cytometer (BD). Data were analyzed using FlowJo software (Tree Star).

### CyTOF analysis

Cytometry by time of flight (CyTOF) allows the simultaneous detection of multiple markers to enable comprehensive immune cell profiling. Briefly, single-cell suspensions were prepared. On the day of CyTOF staining, the single-cell suspensions were stained with metal-tag viability dye for 5 minutes and washed with Cell Staining Buffer (Standard BioTools), followed by staining of surface markers and intracellular markers separately. Cells were then stained with Cell-ID Intercalator-Ir (Standard BioTools) at 4 °C overnight. The next day, cells were washed and prepared for acquisition with Helios (Standard BioTools). Data were collected at the Houston Methodist Research Institute Immunomonitoring Core and analyzed by Cytobank. Raw data were normalized, beads and dead cells gated out, and CD45+ cells were selected to perform t-SNE analysis. Subsequent downstream analysis of comprehensive immune cell characterization was based on a guided gating strategy.

### Statistical analysis

Where appropriate, One-way ANOVA with *post hoc* testing and nonparametric tests (Sign test, Wilcoxon signed-ranks test, and Kruskal–Wallis test) were applied as indicated in figure legends.

All *in vitro* assays were performed at least in triplicate. Fourteen to Fifteen mice per group were used for the *in vivo* studies. A two-tailed Student’s t-test was used to evaluate differences between the controls and each treatment group. P-values < 0.05 were considered statistically significant.

## Results

### A strong immunogenicity induced by TNBCvax vaccine in wild-type non-tumor bearing mice

TOP2A is highly expressed in both human and mouse TNBC tumor tissues and M6 cell lines (12). In this study, we confirmed the expression levels of HIF-1α, IGF-1R and TOP2A proteins in mammary adenocarcinomas tissue. We assessed the expression levels of HIF-1α, IGF-1R and TOP2A proteins in the mammary gland tissues of 16-week-old C3(1)/Tag and wild-type mice using immunohistochemistry staining, as presented in **Figure 1**. Through IHC, we confirmed that HIF-1α and IGF-1R are more highly expressed in the TNBC tumors of C3(1)/Tag mice compared to the mammary gland tissues of wild-type mice.

Next, we evaluated the immunogenicity of single-antigen and a new multi-antigen, multi-peptide vaccine (TNBCvax) that targets three tumor antigens. The vaccines were first administered with CpG adjuvant to immunocompetent, tumor-naïve mice to assess immunogenicity according to the timeline shown in **Figure 2A**. IFN-γ ELISPOT assays showed that IFN-γ-secreting T cells in mouse spleens were significantly increased in the single-antigen vaccinated groups, with the highest increase observed in the TNBCvax group (p<0.0001) (**Figure 2B**), while IL-10 activity remained low across most vaccinated groups (**Figure 2C**). According to the IFN-γ and IL-10 ELISPOT assay result, the TNBCvax exhibited the highest overall immunogenicity compared to each single antigen vaccine.

### TNBCvax generated the strongest anti-tumor efficacy in a syngeneic TNBC mouse model

We tested the immune-preventive anti-tumor efficacy of TNBCvax in a syngeneic tumor graft model using the mammary tumor M6 cell line in C3(1)/Tag-REAR mice. C3(1)/Tag-REAR mice were divided into six groups and received vaccinations according to the timeline presented in **Figure 3A**. As shown in **Figure 3B**, the TNBCvax group (a combination of all three antigens) and the individual IGF-1R, HIF-1α, and TOP2A vaccination groups showed significantly reduced tumor growth rates compared to the CpG control group. At the experimental endpoint of 57 days, the average tumor size in the CpG group was 978.2 mm^3^, whereas it was significantly reduced to 318.1 mm^3^ in the TOP2A group (p<0.0001), 430.9 mm^3^ in the IGF-1R group (p<0.0001), 315.8 mm^3^ in the HIF-1α group (p<0.0001), and 220.8 mm^3^ in the TNBCvax group (p<0.0001).

We also tested the immune responses of single antigen vaccines *vs*. TNBCvax by IFN-γ and IL-10 ELISPOT assays on freshly isolated splenocytes at the experimental endpoint of 57 days. All individual IGF-1R, HIF-1α, TOP2A vaccines and TNBCvax were highly immunogenic as determined by the IFN-γ ELISPOT counts (**Figure 3C**) as compared to low IL-10 ELISPOT responses (**Figure 3D**), with the TNBCvax eliciting the highest immune response. These findings indicated that immune correlates of antitumor efficacy observed with individual single antigen vaccines and TNBCvax were antigen-specific Th1 immunity.

### TNBCvax significantly reduced breast cancer development in C3(1)/Tag transgenic mice

We next evaluated the efficacy of TNBCvax and each individual antigen vaccine in a GEM model of TNBC, C3(1)/Tag transgenic mice. The C3(1)/Tag model is characterized by well-defined tumor progression and frequently used as a highly valuable model for studying molecular changes associated with basal TNBC and for preclinical therapeutic applications. Female C3(1)/Tag transgenic mice develop mammary adenocarcinomas in a predictable manner, exhibiting transitional lesions similar to DCIS in human breast cancer development (21).

Eight-week-old female C3(1)/Tag mice were vaccinated with the TNBCvax following the schedule depicted in **Figure 4A**, with three initial doses given biweekly followed by monthly boosters. Tumor development and progression were monitored in these mice (**Figure 4B**). The results demonstrate that TNBCvax significantly inhibits and delays tumor development compared to the CpG group and other single vaccine-treated groups, including HIF-1α, IGF-1R, and TOP2A. Individual tumor growth curve analysis corroborated the slower tumor progression with smaller tumor volumes in the TNBCvax group, as compared to the other groups. Tumor volumes were significantly reduced in the groups vaccinated with HIF-1α, TOP2A and TNBCvax, as illustrated in **Figure 4C**. At the experimental endpoint, the average tumor volume was markedly lower in the vaccine-treated groups compared to the CpG group. Specifically, the average tumor size was 3,256.5 mm³ in the CpG group, while it was reduced to 1,649.7 mm³ in the TOP2A group (p<0.01), 2,242.5 mm³ in the IGF-1R group, 1,703.2 mm³ in the HIF-1α group (p < 0.05), and down to 361.7 mm³ in the TNBCvax group (p<0.0001).

Further, tumor weight measurements at the experimental endpoint of 24 weeks confirmed a significant reduction in tumor weight in the TNBCvax group compared to the CpG control group (**Figure 4D**). The TNBCvax groups also demonstrated a notable decrease in tumor weight relative to the HIF-1α and IGF-1R groups. Specifically, the average tumor weight at the experimental endpoint was 1.96 g in the CpG control group, whereas it was reduced to 1.14 g in the TOP2A group, 1.58 g in the IGF-1R group, 1.47 g in the HIF-1α group, and 0.32 g in the TNBCvax group (p<0.001). The results indicate that TNBCvax can significantly reduce breast cancer development in C3(1)/Tag transgenic mice. Liver toxicity was assessed by measuring serum ALT and AST levels in vaccinated mice (**Figures 4E-F**). No significant changes in ALT or AST levels were observed across all vaccination groups compared to the CpG control group. We examined the tissues of major organs, including the brain, kidney, liver, lung, and heart, and found no vaccine-associated toxicities in any of the vaccinated groups (data not shown). Taken together, these results show that TNBCvax can induce robust immune responses and significantly reduce breast cancer development in C3(1)/Tag transgenic mice, without any liver toxicity and cardiac toxicity.

**Figure 5:**
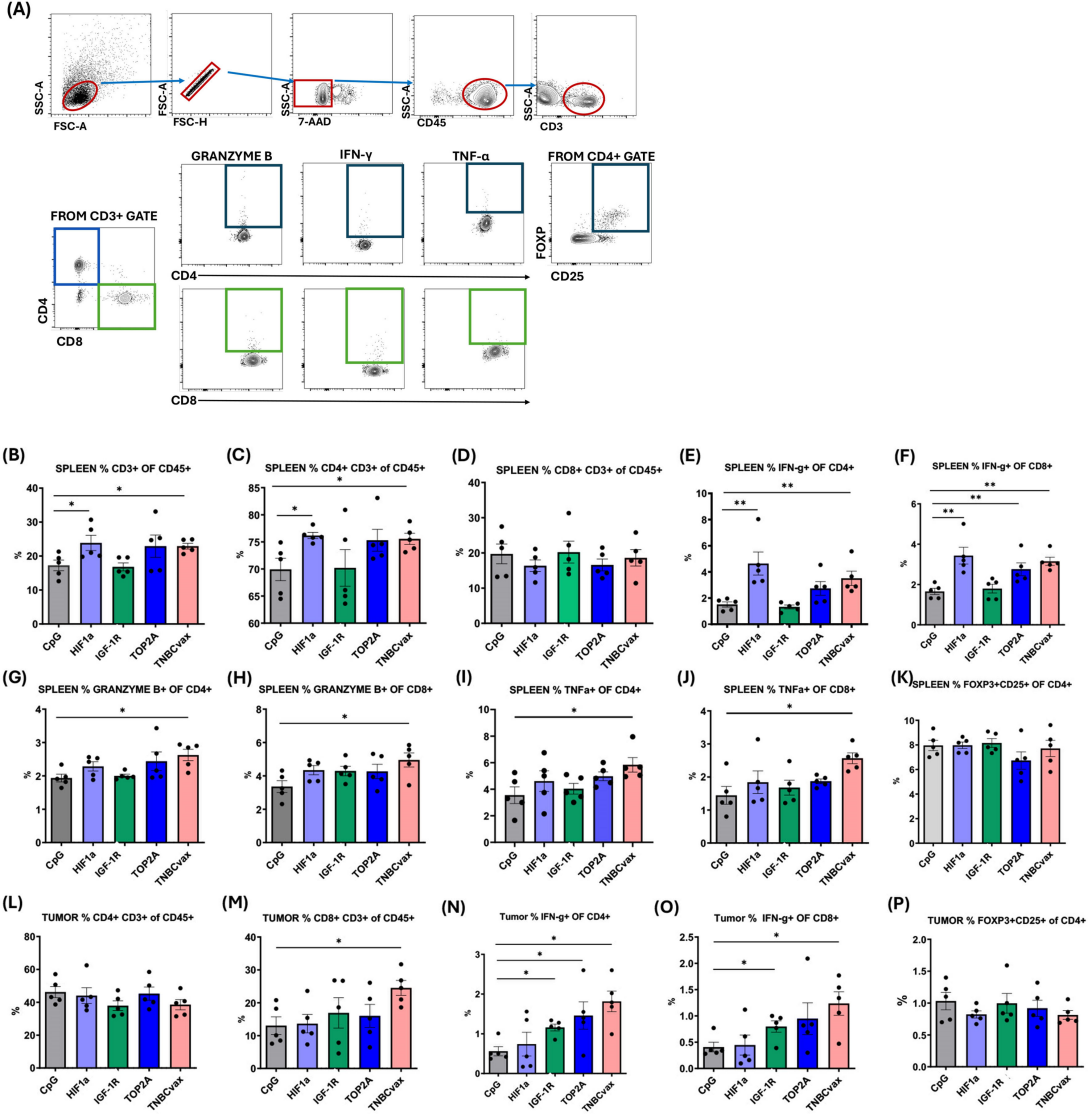
TNBCvax vaccination led to increased antitumor T cell immunity in C3 (1)/Tag transgenic mice. **(A)** Representative images and Flow Gating Strategy. Percentages of **(B)** CD3+ T, **(C)** CD4+ T and **(D)** CD8+ T cells in spleen. IFN-γ expression on **(E)** CD4+ T and **(F)** CD8+ T cells in spleen. Granzyme B expression on **(G)** CD4+ T and **(H)** CD8+ T cells in spleen. TNFα expression in **(I)** CD4+ T and **(J)** CD8+ T cells in spleen. **(K)** Percentages of Tregs in spleen. Percentages of **(L)** CD4+ T and (**M)** CD8+ T cells in tumors. IFN-γ expression in **(N)** CD4+ T and **(O)** CD8+ T cells in tumors. **(P)** Percentages of Tregs in tumors. Data are shown as the mean ± SEM, * p<0.05, ** p < 0.01. The study included six vaccinated groups: CpG group, HIF-1α, IGF-1R, TOP2A groups, and TNBCvax group. A subset of mice (n=5 per group) from the efficacy study (Figure. 4) were used for endpoint flow cytometry analyses. The data were also analyzed statistically with the Sign Test and the Wilcoxon Signed-Ranks Test, Kruskal-Wallis Test.

Flow cytometry results evaluating the immune response following vaccination are presented in **Figure 5**. Cells isolated from the spleens and tumor tissues were stained for surface markers (CD45, CD3, CD4, and CD8) and intracellular markers (granzyme B, IFN-γ, TNF-α, and FoxP3). There was a significant increase in percentages of CD3+ and CD4+ cells in the spleens of mice vaccinated with HIF-1α vaccine, or TNBCvax compared to the CpG control group (**Figure 5B-C**, p<0.05). No significant changes were observed in the percentages of Tregs in the spleen (**Figure 5K**). Functionally activated CD4+ and CD8+ T cells were identified by the intracellular expression of granzyme B, IFN-γ, and TNF-α. The percentages of granzyme B, IFN-γ, and TNF-α in CD4+ and CD8+ T cells were significantly elevated in the spleens of mice vaccinated in the TNBCvax group compared to the CpG control (**Figures 5E-J**, p<0.05). In the tumor tissue, a significant increase in the percentage of CD8+ cells was observed in the TNBCvax group (**Figure 5M**). However, no significant changes were noted in the percentage of CD4+ cells and Tregs in tumor tissues (**Figures 5L, P**). The percentage of IFN-γ in CD4+ T cells was significantly elevated in the tumors of mice vaccinated with the IGF-1R vaccine, TOP2A vaccine, or TNBCvax compared to the CpG control (**Figure 5N**, p<0.05). Similarly, the percentages of IFN-γ in CD8+ T cells were significantly increased in the IGF-1R and TNBCvax groups (**Figure 5O**, p<0.05).

### TNBCvax increased the efficacy of vaccines by enhancing the breadth and potency of the T cell response

**Figure 6:**
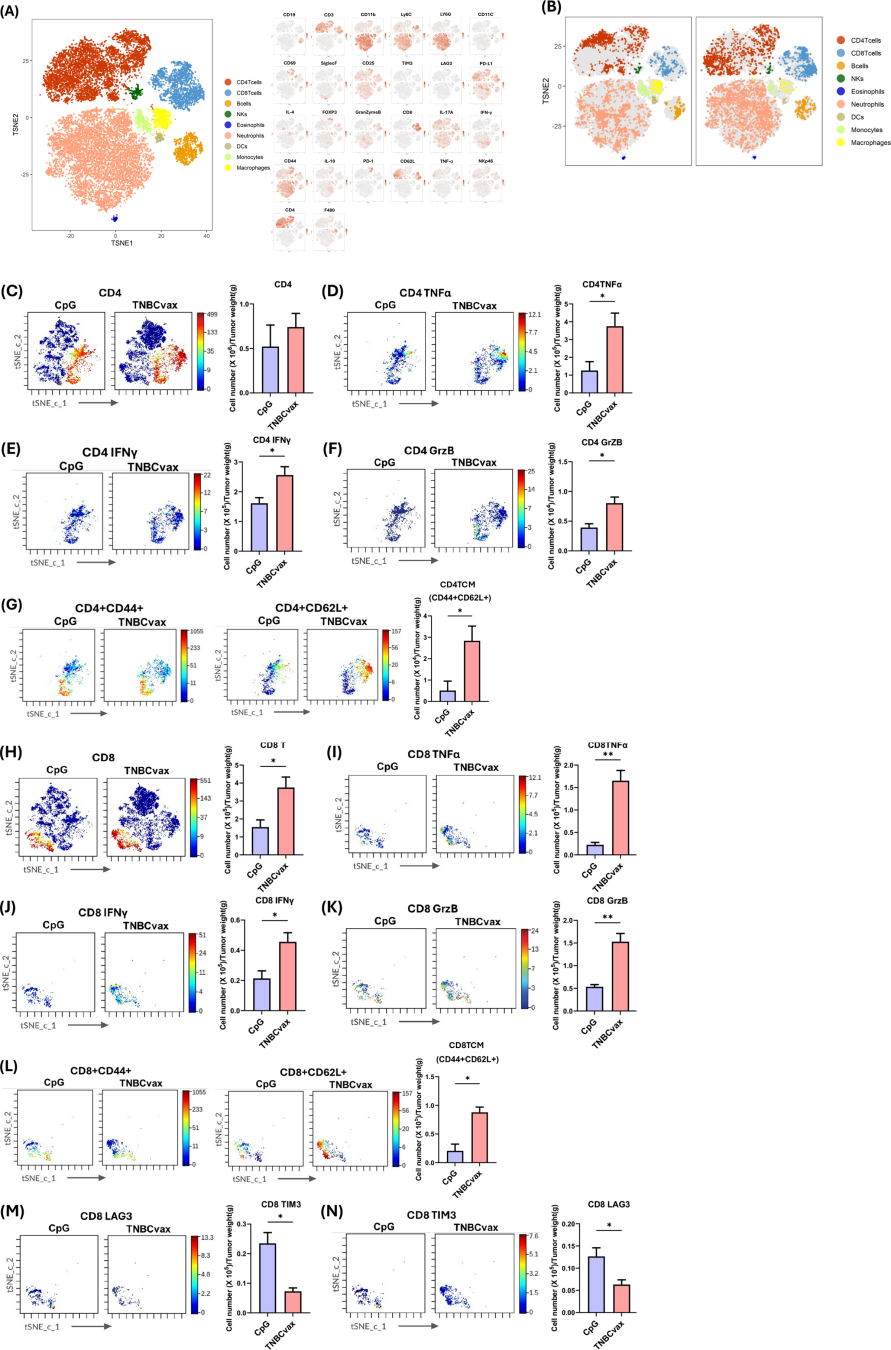
CyTOF analysis of tumor-infiltrating immune cells and functional marker expression affected by TNBCvax vaccination. **(A)** t-SNE plots from CyTOF analysis showing diverse populations of tumor-infiltrating immune cells. The large panel on the left presents the overall t-SNE plot, depicting the diverse populations of tumor-infiltrating immune cells, including CD4+ T cells, CD8+ T cells, B cells, NK cells, eosinophils, neutrophils, dendritic cells (DCs), monocytes, and macrophages. The smaller panels on the right illustrate the expression of each marker used in the 26-marker CyTOF panel for detailed immune profiling across these populations. **(B)** t-SNE plots showing abundance of tumor-infiltrating immune cells for treatment group. **(C-M)** Representative images and quantification of tumor-infiltrating lymphocytes (TILs) per gram of tumor tissue from mice vaccinated with TNBCvax compared to CpG-treated control group. Data are shown as the mean ± SEM (n=3), two-tailed t-test, * p < 0.05, ** p<0.01.

To investigate the immunogenic effects of TNBCvax vaccination, we conducted CyTOF analysis to test tumor infiltrating T cell populations and the expression of related markers. For CyTOF analysis, we performed the same vaccination schedule as in **Figure 5A**. We monitored the development of mammary tumors. Tumor volumes and weight were significantly decreased in TNBCvax group (**Supplementary Figure 1**). The t-SNE plots in **Figure 6A** shows the distribution of immune cell populations within the tumor microenvironment after TNBCvax vaccination including CD4+ T cells, CD8+ T cells, B cells, NK cells, eosinophils, neutrophils, dendritic cells (DCs), monocytes, and macrophages. The distribution of tumor-infiltrating immune cells between CpG group and TNBCvax vaccinated group is shown in **Figure 6B**. The results indicate that TNBCvax vaccination induced significant changes in the tumor immune composition. Especially, the infiltration of CD8+ T cells significantly increased in the tumors of the TNBCvax vaccinated group, which is known to play an important role in the antitumor immune response.

As shown in **Figures 6C-N**, we quantified immune cell infiltration per gram of tumor tissue to evaluate the impact of TNBCvax vaccination. TNBCvax vaccination significantly increased CD8+ T cell infiltration and also upregulated the anti-tumor key effector molecules such as TNFα, IFNγ, and Granzyme B expression (**Figures 6H–K**). These findings indicate that TNBCvax enhances the cytotoxic function of CD8+ T cells in tumor microenvironment. This enhanced pro-inflammatory and cytotoxic activity suggests that TNBCvax vaccination not only induced greater immune cell infiltration into the tumor but also improved their ability to effectively suppress tumor growth. Interestingly, TNBCvax vaccination caused downregulation of LAG-3 and TIM-3 (**Figures 6M, N**). The decrease in LAG-3 and TIM3 suggests that CD8+ T cell exhaustion is alleviated, allowing for sustained antitumor function. Although CD4+ T cells did not significantly change, their functional effector molecules TNFα, IFNγ, and Granzyme B of CD4+ T cells were enhanced, contributing to the observed immunomodulatory effects (**Figures 6C-F**). The central memory T cell (Tcm) populations in both CD4+ and CD8+ subsets were significantly enriched in TNBCvax vaccination group, indicating the induction of a robust, long-lasting memory immune response (**Figures 6G, L**). Collectively, TNBCvax vaccination effectively elevated pro-inflammatory and cytotoxic activity of CD4+ and CD8+ T cells and enhanced central memory T cell responses, and resulting in stronger anti-tumor immune responses compared to CpG control group.

## Discussion

The development and evaluation of TNBCvax, a multi-antigen, multi-peptide vaccine, marks a significant advancement in cancer immunoprevention, particularly for triple-negative breast cancer (TNBC)—a subtype with limited therapeutic options and a poor prognosis. Building on our prior TOP2A vaccine (12), we incorporated HIF-1α and IGF-1R to broaden epitope coverage, enhance immunogenicity of immune cells and reduce the likelihood of antigen-loss–driven immune escape compared to a single-antigen approach. TNBCvax, which targets three key tumor-associated antigens (TOP2A, HIF-1α, and IGF-1R), offers superior tumor prevention and immunogenicity compared to single-antigen vaccines. These antigens were selected for their consistent overexpression in TNBC and their critical roles in tumor pathogenesis. Incorporating multiple antigens addresses the heterogeneity of TNBC and mitigates the risk of antigen-negative variant escape—a limitation observed in single-antigen vaccines such as HER2-targeted approaches in DCIS (10).

Our findings in both the syngeneic M6 tumor graft model and the C3(1)/Tag genetically engineered mouse (GEM) model highlight the efficacy of TNBCvax in reducing tumor development. TNBCvax significantly decreased tumor incidence, volume, and weight while inducing a robust immune response. This response was characterized by increased infiltration of CD4+ and CD8+ T cells, as well as elevated levels of granzyme B, IFN-γ, and TNF-α. These findings suggest that TNBCvax not only recruits immune cells to the tumor microenvironment but also activates them to effectively target and destroy tumor cells. Importantly, TNBCvax exhibited a favorable safety profile, with no significant changes in liver and kidney toxicity markers (ALT, AST, BUN) or evidence of organ toxicity. This safety is crucial for its potential clinical application, as vaccination would target high-risk but otherwise healthy individuals.

Vaccinating with too few antigens will result in selection of antigen loss variants and tumor progression. This has been elegantly demonstrated in animal models by Schreiber and colleagues, indicating loss of single immunodominant CD8 T cell epitope can be adequate for immune escape (26). To induce a broad response that targets a large variety of peptide—MHC I complexes on the tumor cell, vaccines must provide adequate quantities of multiple antigens (17).

Our results demonstrated that the multi-antigen, multi-peptide TNBCvax can increase the efficacy of tumor cell vaccines by enhancing the spectrum and potency of the antigen-specific T cell immune response. TNBCvax significantly enhanced the infiltration and activation of immune cells, particularly CD8+ T cells, within the tumor microenvironment, and functional activation of CD8+ T cells, as evidenced by upregulated expression of key cytotoxic mediators IFN-γ, TNF-α, and Granzyme B. These cytokines play important roles for T cell-mediated tumor cell killing and related immune responses (23).

Furthermore, TNBCvax vaccination also down-regulated the immune checkpoint receptors LAG-3 and TIM-3 on CD8+ T cells, suggesting it obviated CD8 T cell exhaustion. The expressions of LAG-3 and TIM-3 are hallmarks of dysfunctional or terminally exhausted T cells in chronic antigen exposure settings (24, 25). The suppression of LAG-3 and TIM-3 indicates that TNBCvax may maintain CD8+ T cells in a more functional and responsive state. Additionally, TNBCvax increased the population of central memory T cells (Tcm) in both CD4+ and CD8+ subsets, indicating a durable immune memory response. Central memory T cells play a pivotal role in sustaining long-term tumor control by enabling continuous immune surveillance and preventing relapses. Although CD4^+^ T cell infiltration was not significantly changed, TNBCvax vaccination enhanced Th1-associated cytokine production (IFNγ and TNFα) which is suggesting a functional shift in CD4^+^ T cell activity that supports overall immune activation and CD8^+^ T cell-mediated tumor control.

In summary, TNBCvax exerts robust immunogenicity with the ability to minimize antigen-negative variant escape. TNBCvax provides a promising immunotherapeutic strategy capable of orchestrating a coordinated antitumor immune response, improving both CD8+ cytotoxicity and CD4^+^-mediated immune modulation while alleviating immunosuppressive features within the tumor microenvironment. The successful development of TNBCvax could offer a much-needed option for high-risk individuals, potentially reducing the incidence and mortality associated with this aggressive breast cancer subtype.

## Data Availability

The datasets presented in this study can be found in online repositories. The names of the repository/repositories and accession number(s) can be found in the article/supplementary material.

## Supplementary material

Supplementary Figure 1
**(A)** Experimental design and timeline of vaccine administration for CyTOF analysis. **(B)** Tumor growth for the different treatment groups, CpG (n=15) and TNBCvax (n=15), **(C)** Tumor volumes. Palpable tumor diameters were measured at 24 weeks of age. **(D)** Weight of each tumor was taken at the time of sacrifice mice. Data are shown as the mean ± SEM, two-tailed t-test, *** p<0.0001.
