# Supplementary figures and images for: Immunoprevention of triple-negative breast cancer with a novel multivalent vaccine
Source: Front Immunol. 2025 Sep 3;16:1638526. doi: 10.3389/fimmu.2025.1638526 (PMC12440889; doi:10.3389/fimmu.2025.1638526)

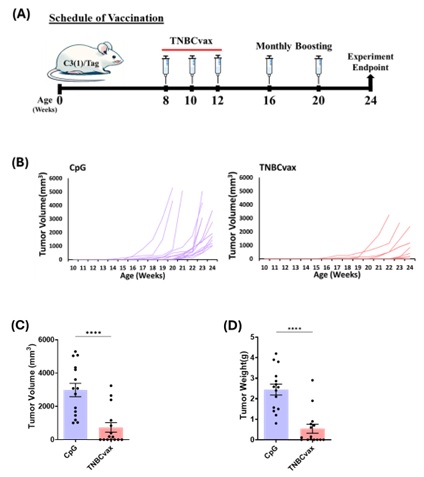

Supplement: Supplementary Figure 1 — (A) Experimental design and timeline of vaccine administration for CyTOF analysis. (B) Tumor growth for the different treatment groups, CpG (n=15) and TNBCvax (n=15), (C) Tumor volumes. Palpable tumor diameters were measured at 24 weeks of age. (D) Weight of each tumor was taken at the time of sacrifice mice. Data are shown as the mean ± SEM, two-tailed t-test, *** p<0.0001. [file Image1.jpeg]
